# Genome-Wide Methylation Patterns in Androgen-Independent Prostate Cancer Cells: A Comprehensive Analysis Combining MeDIP-Bisulfite, RNA, and microRNA Sequencing Data

**DOI:** 10.3390/genes9010032

**Published:** 2018-01-11

**Authors:** Yumin Wang, Tingting Qin, Wangqiang Hu, Binghua Chen, Meijie Dai, Gang Xu

**Affiliations:** Department of Laboratory Medicine, The First Affiliated Hospital of Wenzhou Medical University, ShangCai Village, Ouhai District of Wenzhou, Wenzhou 325000, China; wangyumin@wmu.edu.cn (Y.W.); tingting_qin1999@sina.com (T.Q.); wangqian_hu@sina.com (W.H.); chenbinghua1987@163.com (B.C.)

**Keywords:** androgen-independent, prostate cancer, MeDIP-bisulfite sequencing, microRNA, time-course

## Abstract

This study aimed to investigate the mechanisms underlying the development of the androgen-independent phenotype in prostate cancer. Methylation patterns were detected in androgen-independent and androgen-dependent lymph node carcinoma of the prostate (LNCaP) prostate carcinoma cells based on methylated DNA immunoprecipitation-bisulfite sequencing data and differentially methylated regions (DMRs) were identified. Differentially expressed genes (DEGs) and micro RNAs (miRNAs) with DMRs (named MDEGs and MDEmiRNAs) were identified by combining transcriptome and methylation data, and transcription factor (TF)-DEGs with DMRs in promoter (PMDEGs) and MDEmiRNA-MDEGs networks were constructed. Furthermore, a time-course analysis of gene transcription during androgen deprivation was performed based on microarray data and DMRs, MDEGs, and DEmiRNAs were validated. In total, 18,447 DMRs, 3369 MDEGs, 850 PMDEGs, and 1 MDEmiRNA (miR-429) were identified. A TF-target network (94 PMDEGs and 5 TFs) and a miRNA–target network (172 MDEGs and miR-429) were constructed. Based on the time-course analysis of genes in the networks, *NEDD4L* and *PBX3* were targeted by *SOX5*, while *GNAQ*, *ANLN*, and *KIF11* were targeted by miR-429. The expression levels of these genes and miR-429 were confirmed by quantitative real-time polymerase chain reaction. Additionally, 109 DMRs were confirmed using additional public datasets. The regulatory pathways *SOX5*-*NEDD4L/PBX3*, miR429-*GNAQ/ANLN*—*RHOA*, and miR429-*ANLN*—*KIF11* may participate in the progression of the androgen-independent phenotype in prostate cancer.

## 1. Introduction

Prostate cancer (PC) is one of the leading causes of cancer death in males aged more than 60 years. There are estimated to have been 180,890 new cases of PC and 26,120 deaths caused by PC in the United States in 2016, accounting for 21% of total new cancer cases and 8% of total cancer deaths in males [1]. Currently, androgen ablation therapy (AAT) is a front-line therapy for PC. However, this therapeutic method would fail if the PC developed an androgen-independent phenotype resulting in castration-resistant PC (CRPC), which is a primary cause of refractory and lethal PC [2]. 

Previous studies have elucidated several molecular mechanisms underlying the development of the androgen-independent PC phenotype. Androgen receptor (AR) has been considered a driver of therapeutic resistance in PC via its aberrant activation [3,4] or the presence of AR gene mutations (e.g., AR F876L) or variants (e.g., ARv567es and AR-V7) [4,5]. Furthermore, PC stem cells, which are independent of androgens for survival and have the abilities of paracrine and autocrine androgen biosynthesis and overexpression of antiapoptotic proteins (e.g., B-cell lymphoma 2 (Bcl-2) and glucocorticoid receptor), can also promote resistance to AAT [4,6,7,8]. These genes or proteins that are involved in resistance to AAT are likely to participate in the development of the androgen-independent phenotype. In one of our previous studies, we identified eight novel fusion genes, 315 alternative splicing events, and 788 differentially expressed genes (DEGs) in an androgen-independent PC cell line, lymph node carcinoma of the prostate (LNCaP)-AI-F [9]. Additionally, microRNAs (miRNAs) have also been reported to be associated with the development of the androgen-independent phenotype. Several miRNAs, such as miR-1205, miR-373, miR-21, and miR-29a, are abnormally expressed in androgen-independent PC cells, compared to androgen-dependent PC cells [10,11,12,13]. Moreover, DNA methylation has been found to occur during PC progression and frequently occurs in CRPC [14]. Based on the Panomics gene array system (Panomics, Redwood, CA, USA), one previous study identified that the methylation patterns of several genes (e.g., *CASP8*, *CD14*, and cyclin dependent kinase inhibitor 2A) were significantly different between androgen-sensitive/dependent (LNCaP) and androgen-independent (DU145 and PC3) cells [15]. However, the global distribution of aberrant methylation patterns in the genome of androgen-independent PC cells remains unclear. There has been no comprehensive analysis combining RNA-sequencing, miRNA-sequencing, methylation-sequencing, and microarray data to investigate the global methylation patterns in the genome of androgen-independent PC cells.

Methylated DNA immunoprecipitation sequencing (MeDIP-seq) is a quantitative high-throughput DNA sequencing approach to identify methylated sites with low DNA concentrations based on methyl-DNA immunoprecipitation with an antibody against 5-methylcytosine (5mC) [16]. However, MeDIP-seq is unable to identify individual 5mC sites in captured reads or distinguish un-methylated reads captured by 5mC antibodies due to its non-specific binding, which increases the rate of false positives when detecting methylatedCGs (mCGs) [17]. Whole-genome bisulfite sequencing, the most powerful and complete strategy for the quantitative genome-wide detection of 5mC at a single-base resolution [17], has been reported to reduce the rate of false positives in MeDIP-seq [18]. Thus, a novel method, MeDIP-bisulfite sequencing (MB-seq), is generated by combining the MeDIP-seq protocol with bisulfite treatment. This method can detect the methylation status of each CpG and has a lower rate of false positives than comparable methods, showing more robust sensitivity and specificity for detecting methylatedCpGs (mCpGs) than MeDIP-seq and cytosine methylome sequencing (MethylC-seq) [18]. In MB-seq, the immunoprecipitation step enables the enrichment of methylated fragments of DNA, while the bisulfite treatment enables the distinction of methylated cytosines from un-methylated cytosines in the enriched fragments. Furthermore, in MB-seq, all the cytosines in the adapters are hydroxymethylated [18]. MB-seq has not yet been used to study the molecular mechanisms underlying the progression of the androgen-independent phenotype in PC cells. 

In the present study, MB-seq was applied to investigate the global distribution of DNA methylation across the whole genomes of androgen-independent and androgen-dependent PC cells. Furthermore, RNA-sequencing, miRNA-sequencing, methylation-sequencing, and microarray data were comprehensively analyzed in combination with the MB-seq data. These results provide novel insights into the molecular mechanisms underlying the progression of the androgen-independent phenotype in PC cells.

## 2. Materials and Methods

### 2.1. Cell Lines

An androgen-dependent PC cell line, LNCaP, was purchased from the American Type Culture Collection (Rockville, MD, USA; passage 1). This was cultured in a routine medium and split once per week by trypsinization. The routine medium consisted of phenol red-positive F-12 medium (Gibco^®^, Life Technologies, Carlsbad, CA, USA) and 10% (*v*/*v*) fetal bovine serum (FBS) (Biowest, Nuaillé, France). LNCaP was chronically cultured in an androgen-deprived medium to establish an androgen-independent LNCaP line. First, LNCaP cells were cultured in an androgen-deprived medium composed of the phenol red-free dulbecco's modified eagle medium (DMEM)/F-12 medium (Gibco^®^) plus 10% (*v*/*v*) charcoal/dextran-treated FBS (Biowest) for 5 passages. As flutamide was able to competitively inhibit the binding of androgens to AR, LNCaP cells were then cultured in the above androgen-deprived medium plus 10^−7^ mol/L flutamide (Schering-Plough, Kenilworth, NJ, USA) [13] for 105 passages to obtain a complete androgen-independent cell line. The resulting cell line was named lymph node carcinoma of the cancer prostate—artificially induced—flutamide (LNCaP-AI-F) (passage 110), and its androgen-independence was confirmed by detecting cell morphological changes, cell proliferation rate, cell cycle, and the expression of *BCL-*2 and *BAX* in a previous study [13]. Meanwhile, the androgen-dependent LNCaP cells used as the control were cultured in the routine medium for 110 passages. All cells were cultured at 37 °C in an incubator with 5% CO_2_.

### 2.2. MeDIP-bisulfite Sequencing

Genomic DNA was extracted from LNCaP and LNCaP-AI-F cells using the DNeasy^®^ blood and tissue kit (Qiagen, Hilden, Germany). After the verification of DNA integrity and DNA quantification, 1 μg DNA was sonicated into fragments of 100–500 base pairs (bp). Afterwards, the end-repair of DNA fragments, addition of adenine, and ligation to C-hydroxymethylated Illumina multiplexing adapters (adapter 1: 5′-pho-GATCGGAAGAGCACACGTCT-3′; adapter 2: 5′-ACACTCTTTCCCTACACGACGCTCTTCCGATCT-3′) [19] were performed using the TruSeq DNA sample prep kit (Illumina, San Diego, CA, USA). The DNA was then subjected to a standard MeDIP assay [20,21], and MeDIP-enriched DNA was recovered from the resultant Dynabead^®^-MeDIP-DNA mixture using phenol-chloroform and ethanol. Subsequently, MeDIP-DNA was bisulfite-treated twice using the EpiTect^®^ bisulfite kit (Qiagen) and further amplified for 12 cycles with Illumina multiplexing polymerase chain reaction (PCR) primers 1.0 and 2.0 (final concentration: 0.5 mM; Kapa Biosystems, Wilmington, MA, USA) in an 8 × 25 µL reaction system. After purification of the PCR products and gel electrophoresis on a 2% (*w*/*v*) low melting agarose gel, libraries with insert sizes of 270–370 bp were selected and quantified using the Quant-iT™ PicoGreen^®^ double stranded DNA (dsDNA) reagent and kits (Invitrogen, Carlsbad, CA, USA). Afterwards, the DNA was sequenced for 200 cycles on a next-generation sequencing instrument (Illumina Hiseq 2000 with a TruSeq SBS Kit v3-HS) [18]. The raw MB-seq data were deposited in the Sequence Read Archive (SRA) database (http://www.ncbi.nlm.nih.gov/sra) of the National Center for Biotechnology Information (NCBI) with accession number of SRP067471.

### 2.3. Raw Data Preprocessing and Reads Mapping

The raw data were preprocessed using GA pipeline software version 1.3 (Illumina) with the default settings. Sequencing reads were filtered using FASTX-Toolkit (http://hannonlab.cshl.edu/). Bases with a quality assessment score < 10 at the 5′-terminal or continuous “*N*” at the terminals and reads in which more than 20% of bases had a quality assessment score < 20 were removed. Only clean reads were subjected to the subsequent analyses. 

The Bismark software [22], a flexible aligner and methylation caller for bisulfite-seq data, was applied to map the clean reads to the human genome sequence (hg19) that was downloaded from the Genome Bioinformatics Site at the University of California Santa Cruz (UCSC, http://genome.ucsc.edu). The methylation of C in CpG, CHG, and CHH sequences was detected using the Bismark software [22]. The software parameters were set as seed length = 30 and the number of mismatches within seed ≤ 3.

### 2.4. Differentially Methylated Regions Screening

Based on the clean reads, differential methylation patterns were detected between LNCaP-AI-F and LNCaP samples within a window of 50 bp, and the adjacent windows were merged (length of merged window ≤ 2000 bp). A *t*-test was used to assess the significance of differential methylation, and the raw *p*-value was adjusted into a false discovery rate (FDR) using the Benjamini–Hochberg method [23]. Only the regions with FDR < 0.05 and fold change > 1.25 were identified as differentially methylated regions (DMRs).

### 2.5. Annotation and Distribution of Differentially Methylated Regions

The annotation information about the transcription start site, transcription termination site, exon, intron, and repeats were downloaded from the UCSC database. Information about untranslated regions (UTRs) was obtained from UTRdb (http://utrdb.ba.itb.cnr.it/), and information about enhancers was generated from the VISTA Enhancer Browser (http://enhancer.lbl.gov/). Based on the above information, transcription start sites, transcription termination sites, exons, introns, repeats, UTRs, and enhancers that overlapped with the DMRs were identified.

### 2.6. Analysis of Transcription Factor-Binding Motifs in Differentially Methylated Regions -Promoter-Overlapping Regions

Methylation within CpG islands can impede transcription factor (TF)-DNA binding, affecting the transcription of corresponding genes. The DMRs that overlapped with promoters were obtained using the Perl program (https://www.perl.org/), and motif enrichment analysis was performed using the online tool MEME Suite [24]. Afterwards, the enriched motifs were aligned with the TF-binding motifs of vertebrates in JASPAR CORE 2014 (http://jaspar.genereg.net) [25].

### 2.7. Comprehensive Analysis of Differentially Methylated Regions and Transcriptome

The DEGs and differentially expressed miRNAs (DEmiRNAs) between LNCaP-AI-F and LNCaP cells were reported in our previous studies [9,13]. The chromosomal location information of the DEGs, DEmiRNAs, and DMRs, which was obtained from the Ensembl genome database project (http://asia.ensembl.org/index.html), was input into Linux shell programming [26], and the DEGs and DEmiRNAs that overlapped with DMRs were identified and defined as MDEGs and MDEmiRNAs, respectively. As methylation of the TF-binding region in a gene could inhibit the binding of TF to that region, we specifically defined the DEGs with DMRs in their promoters as PMDEGs.

### 2.8. Pathway and Functional Enrichment Analyses of Differentially Expressed Genes with DMRs (MDEGs)

To study the potential functions of MDEGs, the online tool Targetmine (http://targetmine.nibio.go.jp/) was used to perform pathway and functional enrichment analyses based on the KEGG [27], Reactome [28], WikiPathways [29], NCI-PID [30], and Gene Ontology (GO) [31] databases. The *p*-value was adjusted by the Holm–Bonferroni method, and the adjusted *p*-value < 0.05 was set as the cut-off criterion.

### 2.9. Construction of Transcription Factor-Target Network

Promoters of PMDEGs were aligned with TF-binding motifs in the DMR-promoter-overlapping regions identified above using the MotifDb R package [32] (criterion: matching value in pulsewidth modulation algorithm > 85%). Then the corresponding TF-target pairs and Protein-Protein interactions (PPIs) of targets obtained from the STRING database (http://string-db.org/) [33] were used to construct a TF-target network, which was visualized using Cytoscape software [34].

### 2.10. Construction of micro RNA–Target Network

Based on the information of miRNA–target gene pairs in the online database miRDB [35], the target genes of MDEmiRNAs were predicted (criterion: target score > 50), and the MDEGs among these target genes were selected to construct a miRNA–target network, which was visualized by Cytoscape [34].

### 2.11. Time-Course Analysis of Gene Transcription during Androgen Deprivation

As the regulation of gene expression is a dynamic process, it is important to identify and characterize changes in gene expression over time. Arrays collected over a time course allow for the study of the dynamic behavior of gene expression and clustering analysis has been commonly applied to time-course microarray data [36]. GSE8702 [37], a gene transcription profile during the androgen deprivation of LNCaP, was downloaded from the GEO database (http://www.ncbi.nlm.nih.gov/geo/). The raw data for this dataset were generated via the Affymetrix Human Genome U133 Plus 2.0 Array. GSE8702 contained six LNCaP samples cultured in normal medium (control group) and ten LNCaP samples cultured in androgen-deprived medium (deprivation group) for 3 weeks, 1 month, 5 months, 11 months, and 12 months. In our study, samples in the deprivation group were utilized to study the time-course changes in gene transcription during androgen deprivation. After data pre-processing using the Affy package [38], the fuzzy *c*-means clustering method [39] in Mfuzz software [39,40] was utilized to cluster genes based on their messenger RNA (mRNA) levels at the five time-points. The parameters in the clustering analysis were set as minimum standard deviation = 0.5 and score = 0.6, with a higher score representing a closer clustering. Genes in the Mfuzz-clusters were further clustered, and their transcription levels were shown using pheatmap in R [41]. 

### 2.12. Comprehensive Differentially Expressed Genes Analysis of Sequencing Data and Microarray Data

To identify the genes that were consistently up-regulated or down-regulated during androgen deprivation, the genes in the above Mfuzz-clusters were compared with the DEGs identified in a previous study [9], PMDEGs in the TF-target network, or MDEGs in the miRNA–target network. Venn diagrams were drawn using the online software Venny 2.0.2 [42].

### 2.13. Data Validation of the Identified Differentially Methylated Regions and Differentially Expressed micro RNAs

The methylation profiling dataset GSE41701 [43] deposited in GEO was used for the data validation of the above-identified DMRs. This dataset contained seven benign prostate tissue samples, seven primary localized prostate cancer tissue samples, and 6 metastatic CRPC tissue samples. Only the primary localized prostate cancer tissue samples and metastatic CRPC tissue samples were used for analysis. Firstly, DMRs between the primary and CRPC samples in GSE41701 were identified by a previously reported method [43]. Briefly, bisulfite reads were aligned to the bisulfite-converted hg19 reference genome using Bismark software. All samples had bisulfite conversion rates > 99.5%. Differentially methylated CpGs were identified using the Fisher exact test with correction for multiple testing by the Benjamini–Hochberg method. DMRs were defined as regions containing at least five differentially methylated CpGs (FDR = 0.05) with a fold change > 1.25. Afterwards, the DMRs in GSE41701 that overlapped with those in the present study were identified. 

Furthermore, the non-coding RNA profiling GSE55829 was utilized to validate the expression of the above identified DEmiRNAs, which included four xenograft prostate cancer tissue samples at the androgen-dependent stage and four at the CRPC stage. DEmiRNAs between the androgen-dependent stage and the CRPC stage were identified using a previously reported method [13]. Subsequently, the DEmiRNAs in GSE55829 that overlapped with those in the previous study [13] were identified.

### 2.14. Quantitative Reverse Transcription Polymerase Chain Reaction Assay

Total RNA for mRNA expression analysis was extracted and purified from LNCaP-AI-F and LNCaP cells using TRIzol^®^ reagent (Invitrogen) according to the manufacturer’s protocol. Afterwards, total RNA was reverse transcribed using Moloney murine leukemia virus reverse transcriptase (Promega, Madison, WI, USA). After complementary DNA (cDNA) synthesis, mRNA expression levels were measured using SYBR^®^ Green quantitative polymerase chain reaction (qPCR) Supermix (Bio-Rad, Hercules, CA, USA). Furthermore, total RNA for miRNA expression detection was isolated using the mirVana™ miRNA isolation kit (Ambion, Austin, TX, USA) and purified by the miRNeasy^®^ mini kit (Qiagen). miRNA expression levels were determined using the mirVana™ qPCR detection kit (Ambion). Primers were designed using Primer5 software (PRIMER-E, Plymouth, UK). Each PCR was repeated at least three times.

Expression levels of both mRNA and miRNA were analyzed by a CFX96™ real-time detection system (Bio-Rad). Relative gene expression was calculated using the 2^−ΔΔCt^ method [44]. The expression levels of mRNAs were normalized against glyceraldehyde-3-phosphate dehydrogenase and the expression level of the miRNA was normalized against U6 small nuclear RNA.

### 2.15. Statistical Method

The data were presented as the mean ± standard deviation. Statistical analysis was performed using SPSS 19.0 (IBM, Armonk, NY, USA). Differences in the mRNA expression levels of genes and miRNA between LNCaP-AI-F and LNCaP cells were analyzed by two-tailed Student’s *t*-tests and were considered significant if *p* < 0.05.

## 3. Results

### 3.1. Overview of the MeDIP-bisulfite Sequencing Data

The MB-seq analysis of the LNCaP-AI-F and LNCaP cell lines generated approximately 48.0 and 50.6 million reads, respectively. After data filtering, approximately 80% clean reads were uniquely mapped to the human genome. The ratio of total methylated cytosine in LNCaP-AI-F cells was 8.45%, which was a little higher than that in LNCaP cells (8.20%). In addition, the methylation levels in CpG were much higher than those in CHG and CHH in both LNCaP-AI-F and LNCaP cells.

### 3.2. Annotation and Distribution of Differentially Methylated Regions

**Figure 1:**
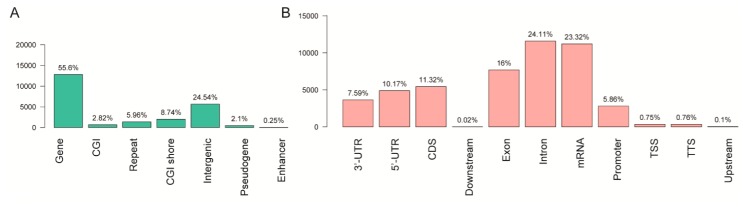

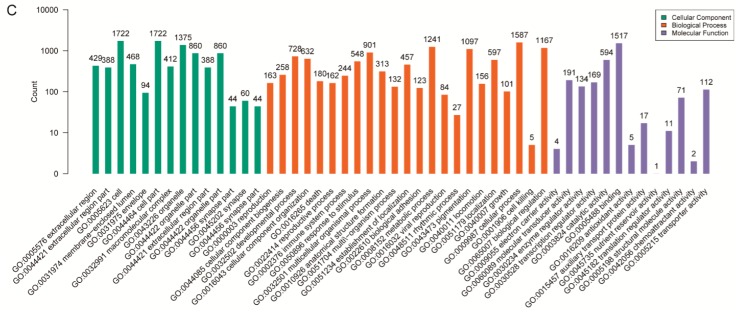
DMRs between lymph node carcinoma of the cancer prostate—artificially induced—flutamide (LNCaP-AI-F) and LNCaP cells. (**A**) Distribution of DMRs by gene structure; (**B**) Distribution of DMRs by functional elements; (**C**) Functions of genes whose promoters overlapped with DMRs. DMRs: Differential methylated regions; CGI: CpG island; UTR: untranslated region; CDS: coding sequence; Downstream: 2 kilobase downstream region of genes; TSS: transcription start site; TTS: transcription termination site; Upstream: 2-kilobase upstream region of genes. In human cells, exons include the CDS and 3′UTR or 5′UTR.

A total of 18,447 DMRs were identified between LNCaP-AI-F and LNCaP cells, including 10,135 hypermethylated and 8312 hypomethylated DMRs in LNCaP-AI-F cells. DMRs were present in 55.6% of genes and 24.54% of intergenic regions (Figure 1A). Additionally, 24.11% of the introns and 23.32% of the mRNAs contained DMRs (Figure 1B).

### 3.3. Transcription Factor-Binding Motifs in Differentially Methylated Regions -Promoter-Overlapping Regions

A total of 2816 DMRs were identified to overlap with the promoters of 2587 genes, and these genes were mainly associated with the functions of cellular processes (GO:0009987) and binding (GO:0005488) (Figure 1C). Additionally, these DMR-promoter-overlapping regions were enriched in ten TF-binding motifs, which were similar to the binding motifs of 20 TFs (Table 1).

### 3.4. Comprehensive Analysis of Differentially Methylated Regions and Transcriptome Data

Based on the obtained DMRs in LNCaP-AI-F cells, and DEGs (4653 up-regulated DEGs and 4074 down-regulated) identified in our previous study [9], a total of 3369 MDEGs were identified, including 1614 up-regulated MDEGs (involving 3840 DMRs) and 1775 down-regulated MDEGs (involving 4925 DMRs). Among the 3840 DMRs, 1989 were hypermethylated and 1850 were hypomethylated in LNCaP-AI-F cells compared with LNCaP cells. Meanwhile, among the 4925 DMRs, 2883 were hypermethylated and 2042 were hypomethylated. Furthermore, among the 2587 genes whose promoters overlapped with DMRs, 381 genes involving 385 DMRs were up-regulated in LNCaP-AI-F cells, and they were defined as up-regulated PMDEGs. In contrast, 469 genes involving 519 DMRs were down-regulated and were defined as down-regulated PMDEGs. Thus, a total of 850 PMDEGs were identified. 

Meanwhile, based on the 86 up-regulated DEmiRNAs and 91 down-regulated DEmiRNAs found in our previous study [13], only one MDEmiRNA was identified (hsa-miR-429). Specifically, hsa-miR-429 was down-regulated in LNCaP-AI-F cells, and it was located within a hypomethylated DMR.

### 3.5. Pathway and Functional Enrichment Analyses of Differentially Expressed Genes with DMRs

The up-regulated MDEGs were significantly enriched in eight pathways and 119 GO terms. The pathways and the top five terms were mainly related to cell cycle, mitotic, nuclear part, and nucleic acid binding (Table 2). Meanwhile, down-regulated MDEGs were significantly enriched in three pathways and 35 GO terms. These pathways and the top five terms were mainly associated with phagocytosis, transport, cytoplasm, transferase activity, and nucleic acid binding (Table 2).

### 3.6. Analysis of the Transcription Factor-Target and micro RNA–Target Networks

**Figure 2:**
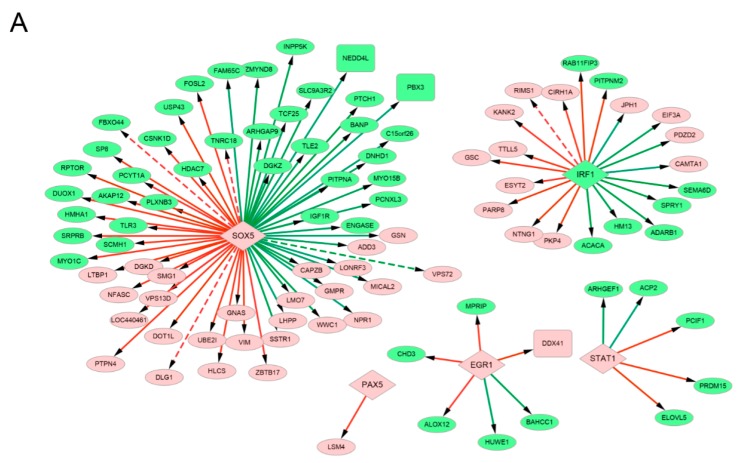

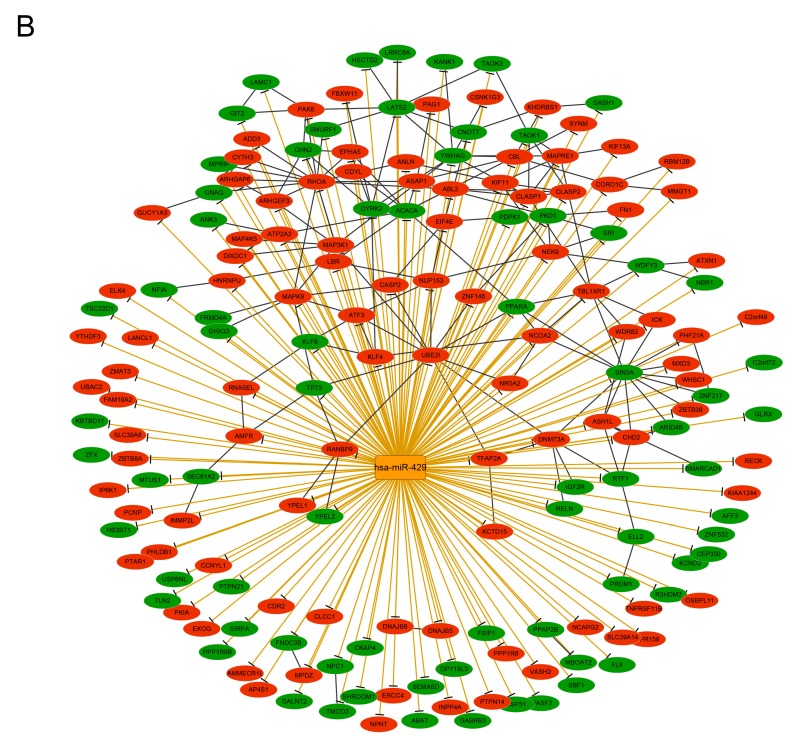
TF target and miRNA–target regulatory networks. (**A**) TF-target network. Red diamond: up-regulated TF; green diamond: down-regulated TF; grey diamond: TF that is not differentially expressed between LNCaP-AI-F and LNCaP cells; red circle: up-regulated PMDEG; green circle: down-regulated PMDEG; red line with arrow: a TF targets a PMDEG and the TF-binding motif overlaps with a hypermethylated DMR; green line: a TF targets a PMDEG and the TF-binding motif overlaps with a hypomethylated DMR; dotted line: the confirmed regulatory relationships by data validation using the GSE41701 dataset; (**B**) bmiRNA–target network. Yellow rectangle: down-regulated MDEmiRNA; red circle: up-regulated MDEG; green circle: down-regulated MDEG; yellow line with T-shape: a MDEmiRNA targets a MDEG; grey line: protein-protein interaction between two MDEGs. TF: transcription factor; miRNA: microRNA; PMDEG: differentially expressed gene whose promoter overlapped with DMR(s); MDEmiRNA: differentially expressed microRNA that overlapped with DMR(s); MDEG: differentially expressed gene that overlapped with DMR(s); DMR: differentially methylated region.

To uncover the potential regulatory relationships among the identified PMDEGs, TFs were predicted from the PMDEGs. The promoters of the 850 PMDEGs were aligned with the ten TF-binding motifs that were identified above (Table 1). Five TFs targeting 94 PMDEGs and 57 protein-protein interactions of PMDEGs were identified, and they were used to construct a TF-target network (Figure 2A). Notably, in the TF-target network, *SOX5* targeted 26 up-regulated PMDEGs and 36 down-regulated PMDEGs.

Furthermore, 768 potential target genes of hsa-miR-429 were predicted and 172 of them were MDEGs, including 98 up-regulated ones and 74 down-regulated ones. In the miRNA–target network of 172 MDEGs and hsa-miR-429 (Figure 2B), *RHOA* interacted with 15 MDEGs. ZEB1, shown in Table 1, was predicted to be a TF targeting hsa-miR-429, and is known to inhibit the translation of hsa-miR-429 [45].

### 3.7. Time-Course Analysis of Gene Transcription during Androgen Deprivation

**Figure 3:**
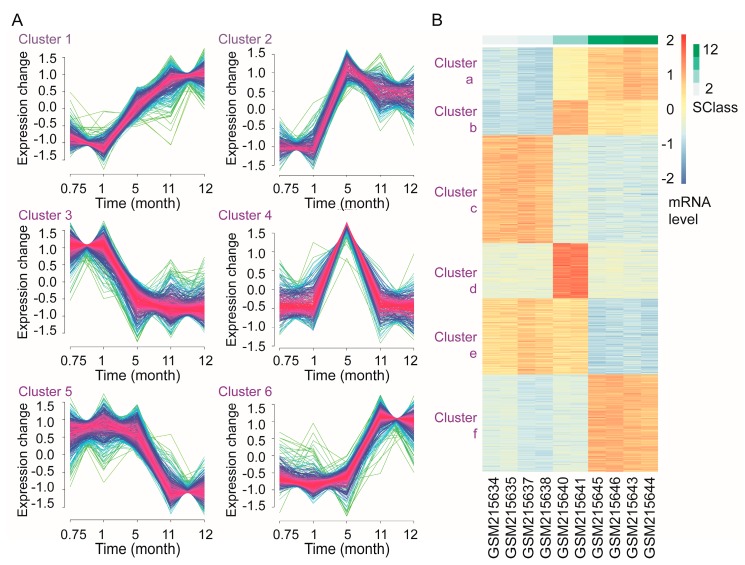
Clustering analyses of genes in LNCaP cells during androgen deprivation. (**A**) Clustering analysis based on Mfuzz software. Red: most close to the clustering center; blue: moderately close to the clustering center; green: least close to the clustering center; (**B**) Hierarchical clustering analysis based on pheatmap software and Mfuzz clusters. Blue: low messenger RNA (mRNA) level; red: high mRNA level. Scale bar represents the duration of androgen deprivation and pheatmap-clusters a, b, c, d, e, and f correspond to Mfuzz clusters 1, 2, 3, 4, 5, and 6, respectively.

Based on the mRNA levels at five time-points, a total of six gene-clusters involving 1367 genes were identified due to their differential expression patterns (Figure 3A). A total of 171 genes in cluster 1, 112 genes in cluster 2, and 313 genes in cluster 6 were up-regulated, whereas 348 genes in cluster 3 and 245 genes in cluster 5 were down-regulated in LNCaP cells during androgen deprivation. Additionally, 178 genes in cluster 4 were first up-regulated and then down-regulated during androgen deprivation. According to the hierarchical clustering analysis, genes in Mfuzz-clusters 1, 2, 3, 4, 5, and 6 were clearly distinguished via pheatmap-clusters a, b, c, d, e, and f (Figure 3B). These results suggested the accuracy of both clustering analyses and the identified genes were defined as time-course genes.

### 3.8. Comprehensive Differentially Expressed Genes Analysis of Sequencing Data and Microarray Data

**Figure 4:**
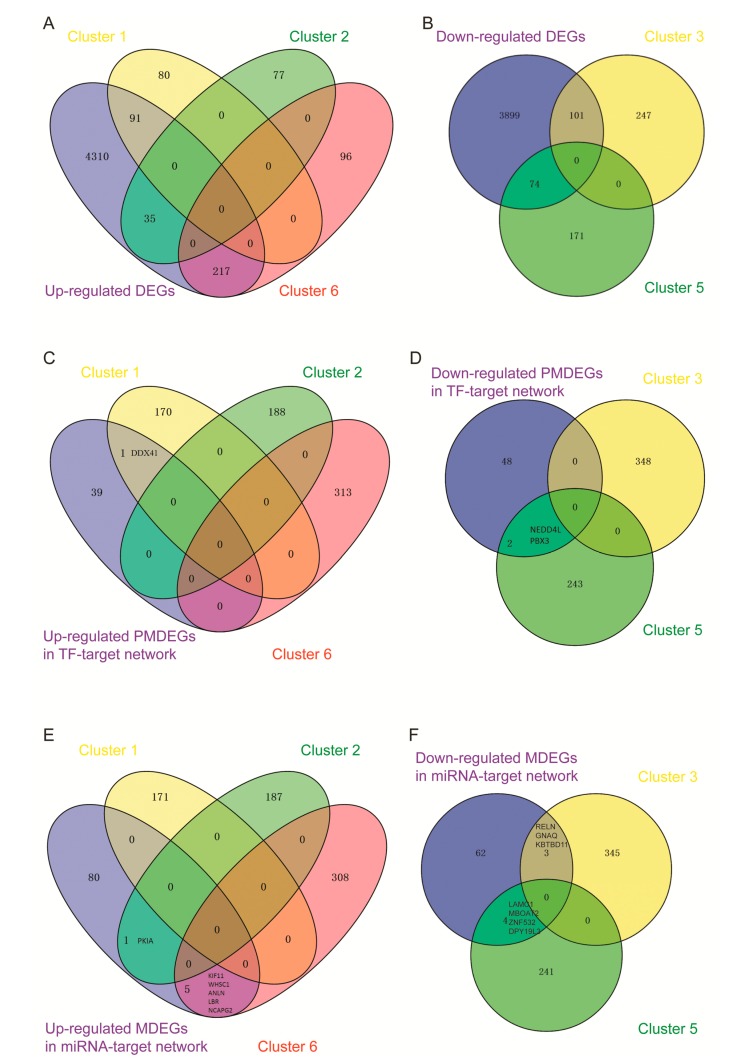
Comprehensive analysis of sequencing and microarray data. (**A**) Genes in clusters 1, 2, and 6 were compared with the 4653 up-regulated DEGs; (**B**) Genes in clusters 3 and 5 were compared with the 4074 down-regulated DEGs; (**C**) Genes in clusters 1, 2, and 6 were compared with the up-regulated PMDEGs in the TF-target network; (**D**) Genes in clusters 3 and 5 were compared with the down-regulated PMDEGs in the TF-target network; (**E**) Genes in clusters 1, 2, and 6 were compared with the up-regulated MDEGs in the miRNA–target network; (**F**) Genes in clusters 3 and 5 were compared with the down-regulated MDEGs in the miRNA–target network. DEGs: differentially expressed genes; TF: transcription factor; miRNA: microRNA; PMDEGs: differentially expressed genes whose promoters overlapped with DMRs; MDEGs: differentially expressed genes that overlapped with DMRs; DMRs: differentially methylated regions.

Genes in clusters 1, 2, and 6 were first compared with the 4653 up-regulated DEGs found in the previous study [9], and respectively 91, 35, and 217 common genes were obtained (Figure 4A). Similarly, genes in clusters 3 and 5 were compared with the 4074 down-regulated DEGs found in the previous study [9], and 74 and 101 overlapped genes were identified, respectively (Figure 4B). These genes were consistently dysregulated in LNCaP cells during androgen deprivation and differentially expressed between LNCaP-AI-F and LNCaP cells. 

Genes in clusters 1, 2, and 6 were compared with the up-regulated PMDEGs in the TF-target network (Figure 2A) and only one common gene *DDX41* was found between cluster 1 and up-regulated PMDEGs (Figure 4C). Genes in clusters 3 and 5 were compared with the down-regulated PMDEGs in the TF-target network (Figure 2A) and only *NEDD4L* and *PBX3* in cluster 5 were among those PMDEGs (Figure 4D). Moreover, both *NEDD4L* and PBX3 were targeted by *SOX5*, namely, *SOX5*-*NEDD4L*/*PBX3*. 

Genes in clusters 1, 2, and 6 were compared with the up-regulated MDEGs in the miRNA–target network (Figure 2B). PKIA in cluster 2, as well as *KIF11*, *WHSC1*, *ANLN*, *LBR*, and *NCAPG2* in cluster 6 were identified as MDEGs in that network (Figure 4E). Genes in clusters 3 and 5 were compared with the down-regulated MDEGs in the miRNA–target network (Figure 2B), and *RELN*, *GNAQ*, and *KBTBD11* in cluster 3, as well as *LAMC1*, *MBOAT2*, *ZNF532*, and *DPY19L3* in cluster 5 were among those MDEGs (Figure 4F). Additionally, both *GNAQ* and *ANLN* interacted with *RHOA* (namely, miR429-*GNAQ*/*ANLN*—*RHOA*) and *ANLN* also interacted with *KIF11* (namely, miR429-*ANLN—KIF11*).

### 3.9. Validated DMRs and Differentially Expressed micro RNAs in Public Datasets

A total of 109 DMRs identified in this study overlapped with those in the dataset GSE41701. The TF targets containing these confirmed DMRs (e.g., *FBXO44*, *VPS72*, and *RIMS1*) are also shown in Figure 2A.

Additionally, a set of 33 up-regulated differentially expressed (DE)miRNAs found in the previous study [13] overlapped with those in GSE55829. None of the down-regulated DEmiRNAs overlapped between those datasets. Strikingly, hsa-miR-429, which was down-regulated in the previous study [13], was up-regulated in GSE55829

### 3.10. Validation of Differentially Expressed Genes with DMRs and Differentially Expressed micro RNAs by Quantitative Real-Time Polymerase Chain Reaction

**Figure 5:**
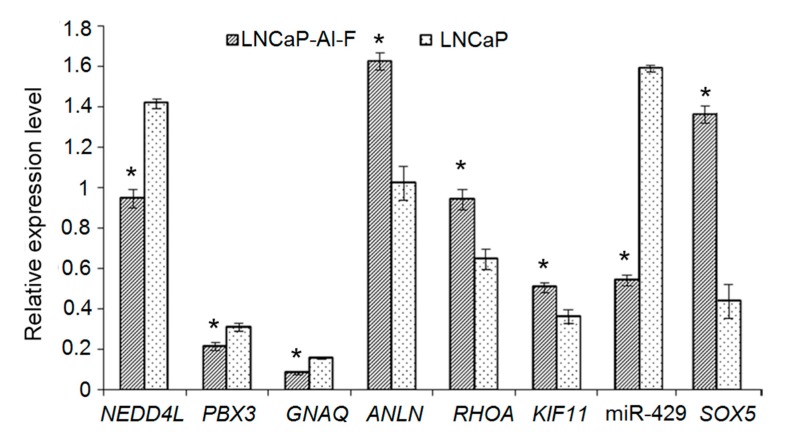
Relative mRNA expression levels of MDEGs and miR-429 determined by quantitative real-time PCR. The expression levels of genes were normalized against glyceraldehyde-3-phosphate dehydrogenase, and the expression level of miR-429 was normalized against U6 small nuclear RNA. * *p* < 0.05. MDEGs: differentially expressed genes that overlapped with differentially methylated regions.

The mRNA expression levels of *NEDD4L*, *PBX3*, and *GNAQ*, as well as hsa-miR-429 were significantly lower in LNCaP-AI-F cells than in LNCaP cells (*p* < 0.05). Furthermore, the mRNA expression levels of *ANLN*, *RHOA*, and *KIF11* were significantly higher in LNCaP-AI-F cells than in LNCaP cells (*p* < 0.05, Figure 5). The results of qRT-PCR were consistent with those obtained via bioinformatics analysis.

## 4. Discussion

In this study, we utilized a novel method, MB-Seq, to detect DNA methylation, and then combined it with RNA-sequencing, miRNA-sequencing, and microarray data to investigate the mechanisms of androgen-independence in PC cells. We identified three potential regulatory pathways, namely *SOX5*-*NEDD4L*/PBX3, miR429-*GNAQ*/*ANLN*—*RHOA*, and miR429-*ANLN*—*KIF11*, which involve the time-course down-regulated PMDEGs of *NEDD4L* and *PBX3*, the time-course down-regulated MDEG of *GNAQ*, the time-course up-regulated MDEGs of *ANLN*, *KIF11*, and *RHOA*, the TF of *SOX5*, and the down-regulated miRNA of hsa-miR-429. 

For the *SOX5*-NEDD4L/PBX3 pathway, *NEDD4L* and *PBX3* were consistently down-regulated in LNCaP cells during androgen deprivation and in androgen-independent LNCaP-AI-F cells compared with those in androgen-dependent LNCaP cells. In addition, they were predicted to be targeted by *SOX5*. *NEDD4L* encodes a ubiquitin ligase, which has been reported to be up-regulated after androgen treatment in LNCaP cells [46]. Decreased expression of *NEDD4L* has been reported to correlate with poor prognosis in gastric cancer patient [47]. Moreover, *NEDD4L* is suggested to be down-regulated in colorectal and lung cancers [48,49]. In the present study, the down-regulation of *NEDD4L* during androgen deprivation and in LNCaP-AI-F cells was consistent with the report by Qi et al. [46]. *SOX5* and DNA methylation may be additional regulators of *NEDD4L* expression during the progression of the androgen-independent phenotype. *PBX3* can regulate genes involved in steroidogenesis and the differentiation of urogenital organs, and steroidogenesis is related to the development of castration-resistant PC. Ramberg et al. reported that *PBX3* is up-regulated in PC tissue and post-transcriptionally regulated by androgen in PC cells in an AR-independent manner [50]. Additionally, *PBX3* is overexpressed in other cancers, such as gastric and colorectal cancers [51,52]. *SOX5* encodes sex-determining region Y-box 5, and its overexpression is associated with the progression and distant metastasis of PC, nasopharyngeal carcinoma, and hepatocellular carcinoma [53,54,55]. SOX9, a homologue of *SOX5*, regulates the expression of AR in PC cells and enhances PC invasion [56,57]. In the present study, the expression levels of *NEDD4L*, PBX3, and *SOX5* were confirmed by qRT-PCR. These findings suggested that *NEDD4L*, PBX3, and *SOX5* may play crucial roles in the development of the androgen-independent phenotype in PC cells. This pathway may be used as a potential therapeutic target in androgen-independent PC. 

For the miR429-*GNAQ*/*ANLN*—*RHOA* pathway, *GNAQ* was down-regulated, whereas *ANLN* and *RHOA* were up-regulated in androgen-independent LNCaP-AI-F cells, compared with those in androgen-dependent LNCaP cells, which was confirmed by qRT-PCR. *GNAQ* mutations have been found in thyroid cancer [58] and *GNAQ/GNA11* mutations are able to initiate human uveal melanoma [59]. Although there are few reports on the function of *GNAQ* in androgen-independent PC, our results suggest the involvement of *GNAQ* in the transition from androgen-dependent LNCaP to androgen-independent LNCaP-AI-F. *ANLN* encodes anillin, an actin binding protein, which has been overexpressed in non–small cell lung and breast cancers, serving as a potential target candidate [60,61]. Importantly, a previous microarray analysis found that *ANLN* was up-regulated in hormone-refractory PC progression [62], which was in agreement with our results. Interestingly, in the present study, both *GNAQ* and *ANLN* were predicted to interact with *RHOA*. *RHOA* encodes a RhoA-GTPase, the activation of which is associated with metastasis in various malignancies. For instance, Kamai et al. [63] found that overexpression of *RHOA* is associated with progression of testicular cancer. The inactivation of RhoA-GTPase mediates the inhibitory effects of *FTY720* on the invasion of androgen-independent PC cells [64], indicating that the activation of *RHOA* may be related to the invasion of androgen-independent PC cells. This result supports our finding that the up-regulation of *RHOA* in androgen-independent LNCaP-AI-F cells coincided with the metastatic property of androgen-independent PC. miR-429 belongs to the miR-200 family, and the miR-200 family of miRNAs are found to be down-regulated in PC cells [65], which is consistent with the results of our study. Interestingly, miR-429 was up-regulated in ovarian and colorectal cancers [66,67], suggesting the different roles of miR-429 in different cancer types. Collectively, *GNAQ*, *ANLN*, and *RHOA* might participate in the androgen-independent progression of PC cells and their expression might be post-transcriptionally regulated by miR-429. They may serve as biomarkers in androgen-independent PC.

For the miR429-*ANLN*—*KIF11* pathway, *ANLN* and *KIF11* were consistently up-regulated in LNCaP cells during androgen deprivation, as well as in androgen-independent LNCaP-AI-F cells. The up-regulation of the two genes in LNCaP-AI-F cells was validated by qRT-PCR. *KIF11*, also known as *KSP* or *Eg5*, encodes the human kinesin Eg5. A high expression level of *KIF11* has been reported in many types of human cancers, such as oral and gastric cancers [68,69]. *KIF11* is required for bipolar spindle formation, which is essential for cell division during mitosis. A previous study found that androgen-independent PC3 cells expressed more Eg5 protein compared with LNCaP cells [70]. These findings suggested that *KIF11* is involved in the development of androgen independence in PC cells and its expression might be regulated by miR-429. Thus, we speculated that the miR429-*ANLN*—*KIF11* pathway may serve as novel prognostic biomarker and therapeutic target for androgen-independent PC.

In the present study, miR-429 was the only identified MDEmiRNA. Although a previous study reported the abnormal expression of miR-429 in LNCaP-AI cells compared to LNCaP cells [13], the methylation status of miR-429 in androgen-independent PC cells has not previously been studied to the best of our knowledge. Strikingly, miR-429 was confirmed by qRT-PCR to be down-regulated in LNCaP-AI-F cells compared with LNCaP cells. However, it was found to be up-regulated in xenograft prostate cancer tissue samples at the CRPC stage compared with the androgen-dependent stage, based on the data validation using GSE55829. We speculate that the differences in sample types (cells were used in the previous study [13] whereas tissues were used in the dataset GSE55829) may contribute to the conflicting results. Further experimental validation in CRPC tissues will be conducted to resolve the conflicting observation.

Despite the aforementioned results, there are several limitations to this study. The regulatory relationships of regulators (e.g., miR-429 and *SOX5*) and their targets need to be confirmed by experiments. Furthermore, the driving effect of DNA methylation on gene expression and the associations between DNA methylation and the development of androgen-independent PC need to be further experimentally investigated. These questions will be addressed in our future studies.

## 5. Conclusions

In conclusion, the regulatory pathways, *SOX5-NEDD4L/PBX3*, miR429-*GNAQ/ANLN*—*RHOA*, and miR429-*ANLN*—*KIF11* may participate in the transition from androgen-dependent LNCaP cells to androgen-independent LNCaP-AI-F cells. Our results may provide novel information for the study of the molecular mechanisms underlying the progression of the androgen-independent phenotype. Those genes and miR-429 may serve as therapeutic targets for delaying the development of androgen-independent PC.

## Figures and Tables

**Table 1:** Transcription factor (TF)-binding motifs in differentially methylated regions (DMR)-promoter-overlapping regions.

Motif	Site	Width	E-Value	Known or Similar Motifs
1	67	30	3.00 × 10^−69^	**IRF1** (MA0050.2)
				**FOXP1** (MA0481.1)
2	1709	5	2.30 × 10^−18^	**STAT1** (MA0137.3)
				**STAT4** (MA0518.1)
				**STAT2**::**STAT1** (MA0517.1)
3	97	29	1.40 × 10^−15^	**ZNF263** (MA0528.1)
				**EGR1** (MA0162.2)
4	1207	5	1.60 × 10^−9^	**ZEB1** (MA0103.2)
				**T** (MA0009.1)
				**SMAD2**::**SMAD3**::**SMAD4** (MA0513.1)
5	1615	5	1.30 × 10^−5^	**GATA4** (MA0482.1)
6	1000	6	2.90 × 10^−5^	**NHLH1** (MA0048.1)
				**MYOG** (MA0500.1)
				**PAX5** (MA0014.2)
7	104	8	1.20 × 10^−3^	**EGR1** (MA0162.2)
8	13	29	1.70 × 10^−3^	**SOX5** (MA0087.1)
9	47	8	4.20 × 10^−3^	**RUNX2** (MA0511.1)
10	510	5	7.50 × 10^−3^	**ARID3A** (MA0151.1)

Letters in bold represent TFs.

**Table 2:** Pathways (all) and functions (top 5) enriched by MDEGs.

MDEGs	Category	Term ID	Term Description	Adjusted *p*-Value	Gene Count
Up	Pathway	R-HSA-69278	Cell Cycle, Mitotic [R-HSA-69278]	4.29 × 10^−6^	85
	Pathway	R-HSA-1640170	Cell Cycle [R-HSA-1640170]	1.91 × 10^−5^	95
	Pathway	R-HSA-453274	Mitotic G2-G2/M phases [R-HSA-453274]	3.59 × 10^−3^	28
	Pathway	R-HSA-1266738	Developmental Biology [R-HSA-1266738]	6.80 × 10^−3^	102
	Pathway	R-HSA-69275	G2/M Transition [R-HSA-69275]	8.10 × 10^−3^	27
	Pathway	vegfr1_2_pathway	Signaling events mediated by VEGFR1 and VEGFR2 [vegfr1_2_pathway]	1.13 × 10^−2^	20
	Pathway	R-HSA-68877	Mitotic Prometaphase [R-HSA-68877]	1.27 × 10^−2^	26
	Pathway	hsa04520	Adherens junction [hsa04520]	1.43 × 10^−2^	20
	GO BP	GO:0000278	mitotic cell cycle [GO:0000278]	4.75 × 10^−13^	153
	GO BP	GO:0007049	cell cycle [GO:0007049]	1.61 × 10^−9^	199
	GO BP	GO:0022402	cell cycle process [GO:0022402]	2.19 × 10^−9^	170
	GO BP	GO:1903047	mitotic cell cycle process [GO:1903047]	6.39 × 10^−9^	119
	GO BP	GO:0090304	nucleic acid metabolic process [GO:0090304]	2.52 × 10^−7^	454
	GO CC	GO:0005622	intracellular [GO:0005622]	1.99 × 10^−18^	1144
	GO CC	GO:0044424	intracellular part [GO:0044424]	1.31 × 10^−17^	1124
	GO CC	GO:0031981	nuclear lumen [GO:0031981]	1.42 × 10^−17^	448
	GO CC	GO:0005654	nucleoplasm [GO:0005654]	2.48 × 10^−17^	392
	GO CC	GO:0044428	nuclear part [GO:0044428]	4.85 × 10^−17^	472
	GO MF	GO:0005515	protein binding [GO:0005515]	1.75 × 10^−12^	1042
	GO MF	GO:0005488	binding [GO:0005488]	1.61 × 10^−10^	1141
	GO MF	GO:0003723	RNA binding [GO:0003723]	3.90 × 10^−7^	198
	GO MF	GO:0003676	nucleic acid binding [GO:0003676]	8.01 × 10^−7^	310
	GO MF	GO:0044822	poly(A) RNA binding [GO:0044822]	3.15 × 10^−6^	172
Down	Pathway	hsa04144	Endocytosis [hsa04144]	4.00 × 10^−4^	52
	Pathway	hdac_classi_pathway	Signaling events mediated by HDAC Class I [hdac_classi_pathway]	4.83 × 10^−3^	20
	Pathway	hsa04666	Fc gamma R-mediated phagocytosis [hsa04666]	9.38 × 10^−3^	24
	GO BP	GO:0016192	vesicle-mediated transport [GO:0016192]	7.26 × 10^−5^	154
	GO BP	GO:0051179	localization [GO:0051179]	9.44 × 10^−5^	511
	GO BP	GO:0006810	transport [GO:0006810]	5.10 × 10^−4^	419
	GO BP	GO:0051234	establishment of localization [GO:0051234]	3.12 × 10^−3^	423
	GO BP	GO:0048518	positive regulation of biological process [GO:0048518]	3.96 × 10^−3^	490
	GO CC	GO:0044424	intracellular part [GO:0044424]	3.47 × 10^−10^	1156
	GO CC	GO:0005622	intracellular [GO:0005622]	2.75 × 10^−8^	1167
	GO CC	GO:0005737	cytoplasm [GO:0005737]	4.37 × 10^−6^	901
	GO CC	GO:0030054	cell junction [GO:0030054]	3.72 × 10^−5^	104
	GO CC	GO:0012505	endomembrane system [GO:0012505]	1.57 × 10^−3^	327
	GO MF	GO:0016773	phosphotransferase activity, alcohol group as acceptor [GO:0016773]	1.58 × 10^−3^	90
	GO MF	GO:0016772	transferase activity, transferring phosphorus-containing groups [GO:0016772]	2.30 × 10^−3^	108
	GO MF	GO:0016301	kinase activity [GO:0016301]	2.52 × 10^−3^	96
	GO MF	GO:0000166	nucleotide binding [GO:0000166]	1.29 × 10^−2^	78
	GO MF	GO:1901265	nucleoside phosphate binding [GO:1901265]	1.40 × 10^−2^	78

MDEGs: differentially expressed genes with differentially methylated region; GO: gene ontology; BP: biological process; CC: cellular component; MF: molecular function; ID: identifier.
